# Exploring the Composition of Blueberry-Based Functional Products: Polyphenolic and Elemental Characterization and Quantification

**DOI:** 10.3390/foods14071210

**Published:** 2025-03-29

**Authors:** Francesca Buiarelli, Maria Presutti, Maria Luisa Astolfi, Carmela Riccardi, Donatella Pomata, Andrea Fricano, Giulia Simonetti, Patrizia Di Filippo

**Affiliations:** 1 Department of Chemistry, “Sapienza” University of Rome, P.le Aldo Moro, 5, 00185 Rome, Italy; francesca.buiarelli@uniroma1.it (F.B.); maria.presutti@uniroma1.it (M.P.); andrea.fricano@uniroma1.it (A.F.); 2 Inail DIT-Via Roberto Ferruzzi, 38, 00143 Rome, Italy; ca.riccardi@inail.it (C.R.); d.pomata@inail.it (D.P.); p.difilippo@inail.it (P.D.F.)

**Keywords:** blueberry, herbal tea, polyphenols, flavonoids, HPLC-MS/MS, ICP-MS, antioxidant activity, human health

## Abstract

**Objectives**: The aim of this study was to provide a comprehensive overview of the nutritional and toxicological aspects of different forms of blueberry products (fresh blueberries, dried blueberries, supplements and herbal teas). **Methods**: Twelve aglycone and glycoside polyphenolic compounds, such as stilbenoids (resveratrol, astringin), flavonols (quercetin, rutin, isoquercitrin, quercitrin, kaempferol), flavanols (catechin, epicatechin), flavanone (hesperitin), flavone (luteolin), and forty chemical elements were analyzed using high-performance liquid chromatography coupled to a mass spectrometer and inductively coupled plasma mass spectrometry. Total phenolic and flavonoid content and antioxidant activity were also evaluated. **Results**: Different distributions of polyphenolic compounds were observed in the blueberry samples, with quercetin and its derivatives, as well as catechin and epicatechin, present in all samples. High concentrations of Ca, K, Mg and P (10–5800 mg/kg) were detected, followed by Fe and Mn at levels below the allowable limits in foods (425 and 500 mg/kg, respectively). The daily intake of polyphenols was quantified, and the estimated daily intake (EDI) was calculated for sixteen elements (including As, Cd, Cu, Fe, Ni, Pb, V and Zn). Hazard quotients (HQs), hazard index (HI) and cancer risk (CR) were assessed for carcinogenic and non-carcinogenic risks associated with the EDI of these elements in food products for both adults and young consumers. For all samples, HI values were below 1, and CR values were within acceptable limits. **Conclusions**: The diversity in polyphenolic profiles and elemental content in blueberry-based products was highlighted by this exploratory study. These findings are valuable for understanding the health benefits and risks of blueberry products.

## 1. Introduction

Functional foods or superfoods are classified by the Food and Agriculture Organization of the United Nations (FAO) as foods that, in addition to providing adequate nutritional principles, contain nutraceutical substances with functions “beyond basic nutrition” [1]. Nutraceuticals, also known as phytochemicals, are bioactive substances that have been scientifically proven to have significant beneficial effects on well-being, promoting human health or reducing the risk of specific diseases. Phytochemicals represent a highly diverse group of compounds, typically characterized by low molecular weight, found in plant-based foods. These compounds can be broadly classified into three major categories: polyphenols, glucosinolates and carotenoids. Polyphenols, which include phenolic acids, flavonoids, lignans and stilbenoids (phenolic compounds derived from stilbene), have attracted significant attention from the scientific community due to their potential health benefits in humans.

Flavonoids, which are secondary metabolites found in plants, are subdivided into various subclasses, including flavones, isoflavones, flavonols, flavanones, flavanonols, flavanols, anthocyanins and chalcones. These subclasses differ in several aspects, such as the number of hydroxyl groups, degree of saturation, the oxidation state, as well as the position and number of attached glycosides (Figure S1A,B) [2].

They are known for their biological and pharmacological properties, such as antioxidant [3], anti-inflammatory [3], antimutagenic [4,5,6], anticancer [7,8,9], antithrombotic [10] and antimicrobial [11] activity. These compounds are abundantly present in a variety of plant-based foods, including red fruits, berries, blueberries and other vegetables [12], as well as in beverages such as herbal tea [13,14]. Among the fruits, wild berries can be one of the best sources of polyphenols in the human diet; in particular, blueberries are an excellent source of these compounds, mainly flavonoids such as anthocyanins, flavonols and flavanols, with high antioxidant capacities compared to other fruits [15,16] and consequent protective biological actions on human health [12]. Due to their high content of antioxidant compounds, they are considered superfoods and are widely used in several products, including food supplements, and herbal products such as herbal teas. These effects establish blueberries as a significant subject of study for the prevention and treatment of chronic diseases, making them exemplary candidates for research in health and nutrition. Moreover, the global market for blueberry-based products is expanding rapidly, driven by consumer demand for natural and health-promoting ingredients. This trend underscores the importance of studying blueberries to innovate and enhance product offerings in the food and health industries [17]. The study of individual polyphenolic compounds and the identification of characteristic profiles in real samples require the use of separative analytical techniques; in particular, HPLC coupled with ultraviolet–visible (UV–VIS) and/or tandem mass spectrometry (MS/MS) detection are the most used for the analysis of specific polyphenolic compounds in food extracts [18]. In addition, scientific research [19] has been focusing over time on the evaluation of polyphenolic content using rapid colorimetric tests, total phenolics content (TPC) and total flavonoids content (TFC), respectively, which allow the quantification of the total content of these compounds without the possibility to perform their specific identification. However, these kinds of tests can be heavily affected by the presence of interferences, which could lead to overestimation of the content of these compounds. Moreover, despite these assays being frequently used, they do not provide any correlation between specific substances and their antioxidant activity. For the antioxidant activity analysis, several methods are used in the literature, mostly based on the reagents used in the tests, such as ferric reducing antioxidant power (FRAP), oxygen radical absorbance capacity (ORAC), cupric reducing antioxidant capacity (CUPRAC), 2,2-Dipheny/L-PicrylHydrazyl (DPPH) and 2,2′-azino-bis((3-eylbenzothiazoline-6-sulfonic acid) (ABTS) [20,21,22,23,24]. In addition, plant-based foods are significant in the human diet because of their content in vitamins and trace elements, which are currently considered to be essential for the normal biological functioning of human beings [25,26]. Another important aspect to consider when discussing natural foods is assessing the presence of toxic or potentially toxic metals that may be present in the final product [25,26]. Metals can be incorporated into organic structures or may result from contamination through natural products, leading to a possible accumulation, due to environmental contamination (air pollution, soil contact) or during the manufacturing process. Furthermore, the effects of various concentrations of heavy metals on plant growth demonstrate that higher concentrations of these metals may inhibit the synthesis of secondary metabolites in plants [27]. In this context, a parallel study of the presence of metals in superfoods could be interesting for evaluating possible ingestion risks for human health. Notably, there are very few studies that conduct comprehensive risk assessments of metals, and these often focus on only a select few metals, neglecting many others [28]. This gap in research highlights the urgent need for more extensive studies that include a broader range of metals to ensure a thorough evaluation of potential health risks.

This research aims to optimize a rapid and efficient analytical method for the detection of polyphenolic compounds, both aglycones and glycosides, and to evaluate the contribution of elements present in various blueberry-based foods to assess potential associated risks. The attention is focused on blueberries, well known to be rich in polyphenols, including flavonoids, known for their antioxidant properties and benefits for cardiovascular and visual health [9].

These include flavanols (catechin, epicatechin), flavonols (quercetin, rutin, isoquercitrin, quercitrin, kaempferol), flavanone (hesperetin), flavone (luteoline) and stilbenoids (resveratrol, astringin). The objective is to provide a reliable and efficient method for profiling polyphenolic content across a diverse range of products. After the solid phase extraction (SPE) optimized by Simonetti et al. [29], HPLC-ESI-MS/MS was used to analyze these compounds in different blueberry-based functional foods, such as fresh and dried blueberries, supplements and herbal teas.

Once the polyphenolic profiles of various blueberry-based products were obtained, the results were compared with the total phenolic content (TPC), total flavonoids content (TFC) and were assessed in terms of antioxidant activity using the DPPH and ABTS methods. The data obtained from TFC and TPC assays allowed us to also carry out a complete evaluation of the intake of flavonoids and polyphenols based on the average daily intake considered reasonable for each sample.

In parallel, all samples were analyzed via inductively coupled plasma mass spectrometry (ICP-MS) to detect chemical elements. A comprehensive nutrient profile was assessed, alongside an evaluation of potential accumulation of toxic or potentially harmful elements. The toxicity of heavy metals, classified as harmful, can induce oxidative stress, inflammation, apoptosis and subsequent pathological alterations in signaling pathways. Subsequently, the elemental results were used to calculate the estimated daily intake of metals (EDI), hazard quotient (HQ), hazard index (HI) and cancer risk (CR) to assess the potential human health risks associated with the ingestion of toxic or potentially harmful elements through blueberry-based food intake. Although this is a preliminary study on a limited number of samples, it sets a benchmark for further research aimed at providing insights into the polyphenolic and elemental composition of various blueberry-based functional products. This study definitively introduces a novel, comprehensive profiling method for polyphenolic content in various blueberry-based products, integrating advanced analytical techniques and parallel elemental analysis to provide a holistic assessment of both nutritional benefits and potential health risks.

## 2. Materials and Methods

The standards used were of high purity:

- Resveratrol (3,4,4′-triidrossi-trans-stilbene; Karlsruhe, Carl Roth GmbH + Co. KG, Mühlburg, Germany);
- Piceatannol (3,3′,4,5′-Tetrahydroxy-trans-stilbene; MCE, MedChemExpress, Monmouth Junction, NJ, USA);
- Astringin (3,3′,4,5′-Tetrahydroxy stilbene 3′-glucoside; MCE, MedChemExpress, Monmouth Junction, NJ, USA);
- Catechin ((+)—Cyanidanol, D-Catechin; MCE, MedChemExpress, Monmouth Junction, NJ, USA);
- Epicatechin ((−) Epicatechin, cis-D-Catechin; MCE, MedChemExpress, Monmouth Junction, NJ, USA);
- Rutin (Quercetin 3-rhamnoglucoside; Karlsruhe, Carl Roth GmbH + Co. KG, Mühlburg, Germany);
- Isoquercitrin (Quercetin 3-β-D-glucoside; Extrasynthese, Genay, France);
- Quercitrin (Quercetin 3-rhamnoside; Karlsruhe, Carl Roth GmbH + Co. KG, Mühlburg, Germany);
- Quercetin (Quercetin hydrate; Karlsruhe, Carl Roth GmbH + Co. KG, Mühlburg, Germany);
- Kampferol (3,4′,5,7-Tetrahydroxy flavone; MCE, MedChemExpress, Monmouth Junction, NJ, USA);
- Hesperetin (3′,5,7-Trihydroxy-4′-methoxy flavanone; Extrasynthese, Genay, France);
- Luteolin (3′,4′,5,7-Tetrahydrox flavone; Karlsruhe, Carl Roth GmbH + Co. KG, Mühlburg, Germany);
- Multi-element standard solution for ICP-MS calibration (1.000 ± 0.005 mg/L As, Al, Ba, Be, Bi, Cd, Ce, Co, Cr, Cs, Cu, Ga, La, Li, Mn, Mo, Nb, Ni, Pb, Rb, Sb, Se, Sn, Te, Ti, Tl, U, V, W and Zr; 10.00 ± 0.05 mg/L Fe and Zn; 50.00 ± 0.25 mg/L P and Si; 55.00 ± 0.25 mg/L B and Sr; 500.0 ± 2.5 mg/L K, Mg and Na; 1000 ± 5 mg/L Ca and S in 5% HNO_3_, VWR International S.r.l., Milan, Italy);
- Single standard solution for ICP-MS internal standards (1000 ± 2 mg/L Y, Panreac Química, Barcelona, Spain; 1000 ± 5 mg/L Sc, Rh, In and Th, Merck KGaA, Darmstadt, Germany) to monitor matrix effects and sensitivity drifts;
- Multi-standard stock solution of Ba, Be, Ce, Co, In, Pb, Mg, Tl and Th (10.00 ± 0.05 mg/L, Spectro Pure, Ricca Chemical Company, Arlington, TX, USA) for testing ICP-MS performance;
- NIST 1643f trace elements in water (National Institute of Standards and Technology, NIST; Gaithersburg, MD, USA) were used to assess the elemental analysis accuracy.

The solvents, reagents and material used were of high purity:

- Nitric acid (HNO_3_, 70%, super-pure) (Carlo Erba Reagents S.r.l., Milal, Italy);
- Methanol (CH_3_OH, MeOH) (Carlo Erba Reagents, Milan, Italy);
- Acetonitrile (AcN) (Romil-UpSTM Ultra Purity Solvents, London, UK);
- Formic acid (HCOOH) (Carlo Erba Reagents, Milan, Italy);
- Water Milli-Q (Millipore Corporation, Burlington, MA, USA);
- Ethanol (CH_3_CH_2_OH, EtOH) (Carlo Erba Reagents, Milan, Italy);
- Sodium nitrite (NaNO_2_) (Carlo Erba Reagents, Milan, Italy);
- Aluminum trichloride (AlCl_3_) (Carlo Erba Reagents, Milan, Italy);
- Sodium hydroxide (NaOH) (Carlo Erba Reagents, Milan, Italy);
- Sodium carbonate (Na_2_CO_3_) (Sigma-Aldrich Co., Saint Louis, MO, USA);
- Folin–Ciocâlteau reagent (Sigma-Aldrich Co., Saint Louis, MO, USA);
- Sodium persulfate (NaS_2_O_8_) (Carlo Erba Reagents, Milan, Italy);
- Sodium hydrogen phosphate (Na_2_HPO_4_) (Sigma-Aldrich Co., Saint Louis, MO, USA);
- 2,2′-azino-bis (3-eylbenzothiazoline-6 sulfonic acid (ABTS) (Sigma-Aldrich Co., Saint Louis, MO, USA);
- 2,2-Dipheny/L-picrylhydrazyl (DPPH) (Sigma-Aldrich Co., Saint Louis, MO, USA).

Vacuum Manifold, Air Cadet^®^ was used for the elution of cartridges; it consists of a glass container inside which collection tubes are placed and in which a mild vacuum is generated.

Filtration was performed using 0.45 µm filters (Millex HV, Millipore, MA, USA) and 0.22 µm filters (Millex^®^-CV 0.22 µm) from Phenomenex^®^ (Castel Maggiore Bologna, Italy).

The investigated HPLC-MS/MS standard solutions, filtered before use, were prepared at concentrations of 1 mg/mL, each dissolving the pure compounds in methanol stored at −20 °C in the dark. The HPLC-MS/MS working solutions were prepared daily by diluting the primary solution according to the desired concentration (40 μg/L–5 μg/mL) and stored at 4 °C in amber vials.

### 2.1. Samples

Samples of fresh and dried blueberries and blueberry-based herbal teas were chosen as foods rich in polyphenols and purchased from various supermarkets and/or retail stores. In particular, the fresh and dried blueberry products analyzed were obtained as distinct samples in their final form. The objective was to assess realistic consumption of commercially available products. These were two separate products, not the same product subjected to a drying process. The selection of different products was aimed at considering the possible edible forms of blueberry-based products available in the market. In addition, blueberry-based dietary supplements, widely used today as a source of nutrients according to the Ministerial Guidelines (LGM) (Directive 2002/46/EC) [30], were tested. Despite the selection of a limited number of samples, this approach aimed to evaluate a diverse range of blueberry-based products that consumers can easily access and incorporate into their diets. This methodology was designed to reflect the actual intake of polyphenols from these products in the consumer’s daily nutrition.

Table 1 shows the samples tested and their relative composition.

### 2.2. Sample Pre-Treatment

After being weighed, fresh, dried blueberries and blueberry-based supplements were treated with hot water, and the composition of the extracted solution obtained was compared with the herbal tea.

#### 2.2.1. Fresh Blueberries

Fifteen blueberries were weighed and subsequently homogenized. A small aliquot (3.15 g) was then diluted in 10 mL of hot water. After 10 min of sonication in an ultrasonic bath (Ultrasonic Cleaner, Soltec^®^, Milan, Italy), the supernatant was separated from the bottom, centrifuged for 5 min and filtered with a 0.45 μm filter.

#### 2.2.2. Dried Blueberries

Four dried blueberries (~2.74 g) were weighed and ground in a mortar, and the resulting extract was diluted in 10 mL of hot water.

After 10 min of sonication in an ultrasonic bath, the supernatant was separated from the bottom, centrifuged for 5 min and filtered with a 0.45 μm filter.

Despite the comparable weight used, all comparisons in this study accounted for the compositional differences due to the content of water in fresh blueberries, about 85–90%, while dried blueberries only had a water content of about 10–15%.

#### 2.2.3. Supplement

One capsule (0.305 g) was ground in a mortar and diluted in 10 mL of hot water. After 10 min of sonication in an ultrasonic bath, the supernatant was separated from the bottom, centrifuged for 5 min and filtered with a 0.45 μm filter. According to the product label, each capsule contains 200 mg of blueberry extract, corresponding to 66% of the total capsule weight.

#### 2.2.4. Herbal Tea

All the herbal teas were prepared by simulating the usual domestic preparation: 300 mL of water was heated at 100 °C, and the herbal tea bag (7 g) was subsequently infused for five minutes, as mentioned on the packaging information. A 10 mL aliquot of herbal tea was taken and filtered with a 0.45 μm filter.

### 2.3. Total Phenolics Content (TPC)

TPC measurement was carried out according to the modified Folin–Ciocâlteau method described by Shannon et al. [22]. A volume of 5 mL of Folin–Ciocâlteau reagent and 10 mL of 20% Na_2_CO_3_ were sequentially added to 1 mL of the pre-treated sample (see Section 2.2). The solution was brought to a volume of 25 mL. After 30 min of incubation at 25 °C, the absorbance at 765 nm was measured using a UV–VIS spectrophotometer (UV–Vis, Varian Cary 50 UV–VIS Spectrometer, Victoria, Australia). The results were calculated using linear interpolation to a calibration curve of gallic acid (200–1000 μg/mL) and expressed in mg of gallic acid/g of sample (mg GAE/g sample).

### 2.4. Total Flavonoids Content (TFC)

TFC measurement was carried out according to the modified AlCl_3_ assay described by Abdullah et al. [31]. A volume of 1.2 mL of deionized water and 95 μL of 5% NaNO_2_ were sequentially added to 400 μL of the pre-treated sample (Section 2.2). After 5 min, 95 μL of 10% AlCl_3_ was added. After 5 min, 640 μL of a 1 M solution of NaOH was added, and the solution was agitated for 1 min. The solution was left to rest in the dark for 15 min, and its absorbance was measured at 510 nm using a UV–VIS spectrophotometer. The results were calculated using linear interpolation to a calibration curve of quercetin (100–5000 μg/mL) and expressed in mg of quercetin/g of sample.

### 2.5. Total Antioxidant Capacity

#### 2.5.1. DPPH Radical Scavenging Activity

The antioxidant capacity was determined according to the modified DPPH radical scavenging capacity assay described by Chan et al. [32]. A volume of 2925 μL of a DPPH 0.1 mM (prepared by weighing 2 mg DPPH dissolved in 50 mL of ethanol) was added to 75 μL of the pre-treated sample (Section 2.2). The absorbance at 517 nm at time zero was measured using a UV–VIS spectrophotometer. After 30 min of incubation and agitation in darkness at 25 °C, absorbance was measured at the same wavelength.

DPPH radical scavenging capacity was calculated according to Equation (1):

(1) $$\%I_{DPPH}=\frac{\left({Abs}_{t0}-{Abs}_{t30}\right)}{{Abs}_{t0}}\times 100$$

DPPH radical scavenging capacity was expressed in mg of Trolox/g of sample using interpolation to a calibration curve of Trolox (1.25–0.25 mM).

#### 2.5.2. ABTS Radical Cation Decolorization Activity

The antioxidant capacity was determined using the ABTS radical cation decolorization activity assay. The radical cation ABTS•+ was generated by the reaction between a 7 mM solution of ABTS and a 2.45 mM solution of sodium persulfate. The reaction took place in the dark for 12–16 h at room temperature. A volume of 1980 μL of an ABTS•+ solution was added to 20 μL of the pre-treated sample (Section 2.2). The absorbance at time zero was measured at 734 nm using a UV–VIS spectrophotometer. After 6 min, absorbance was measured at the same wavelength.

ABTS•+ radical cation activity was calculated according to Equation (2):

(2) $$\%I_{ABTS}=\frac{\left({Abs}_{t0}-{Abs}_{t6}\right)}{{Abs}_{t0}}\times 100$$

ABTS•+ radical cation activity was calculated using interpolation to a calibration curve of Trolox (1.25–0.25 mM) and expressed in mg of Trolox/g of sample.

### 2.6. Analysis of the Extracted Elements

Fresh, dried blueberries, blueberry-based supplement and blueberry-based herbal tea extracted solutions (10 mL) were acidified with 100 μL concentrated HNO_3_, spiked with internal standards (Y at 5 μg/L, and Sc, Rh, In and Th at 10 μg/L) and analyzed via ICP-MS (820-MS, Bruker, Bremen, Germany) without any further dilution. Blanks and calibration standards were also prepared as the extracted samples. The ICP-MS operating parameters have been described previously [33].

### 2.7. Analysis of Polyphenols

Polyphenols were identified and quantified using a high-performance liquid chromatography system with a high-pressure mixer (Agilent 1260 Infinity II, Waldbronn, Germany) coupled to a triple quadrupole mass spectrometer (Api 2000 Applied Biosystem SCIEX, Waltham, MA, USA) equipped with an electrospray ionization (ESI) interface as the ionization source; the chromatographic system is fitted with a Gemini^®^ C-18 column (150 × 2 mm, 3 μm, 110 Å, Phenomenex, Torrance, CA, USA).

### 2.8. Quality Assurance

All ICP-MS calibration curves were produced over nine calibration levels (including zero) and scored a correlation coefficient of at least 0.999 and good linear relationships across all concentration ranges studied, as verified by the instrument software. The ICP-MS calibration ranges were selected according to the expected concentrations of the elements of interest in the analyzed extracted solutions. The detection and quantification limits (LODs and LOQs, respectively) of each element were calculated as three and ten times the standard deviation of the blank sample, which was the deionized water used for the experiment (10 replicates). The LOD and LOQ values ranged from 0.004 (Tl) to 4000 μg/Kg (Na) and from 0.01 (Tl) to 13,000 μg/Kg (Na), respectively (Table S1). The accuracy (as trueness bias percentage), evaluated using NIST 1643f trace elements in water, was <10% for all the certified elements, while repeatability as a relative standard deviation percentage (RSD%) was <10%. The possible matrix effect and the influence of ICP-MS instrumental drift were checked using an internal standard solution of In, Rh, Sc, Th and Y. For the HPLC-MS/MS analysis, the polyphenol standard solutions were prepared at seven different concentration levels, ranging from 40 µg/L to 5 µg/mL, to assess the linearity of the calibration curves. The instrumental limits of detection (LOD) and quantification (LOQ) were accurately determined with acceptable precision. The intra- and inter-day precision of the method was evaluated by injecting a multi-standard solution three times within a single day and over three consecutive days. The results were expressed as relative standard deviation (RSD); values < 15% can be considered suitable for HPLC-MS/MS multi-analyte methods. Further details on the HPLC-ESI-MS/MS optimized operating conditions and method validation are reported in the Supplementary Materials. The extraction recovery and matrix effect were calculated as reported in the study of Simonetti et al. [29], with some method modifications consisting of the use of methanol as the final eluent (4 mL) and reduced to 1 mL prior to injection in HPLC-MSMS.

### 2.9. Calculation

#### 2.9.1. Polyphenol/Flavonoid Daily Intake

The flavonoid/polyphenol daily intakes (FDI/PDI) of the selected food and supplement samples were calculated in agreement with Equation (3):

(3) $$FDI(PDI)=TFC(TPC)\left(\frac{mg}{g}\right)\times AID\left(\frac{g}{day}\right)$$

where TFC or TPC is the value of total flavonoid or polyphenol content measured by the TFC/TPC assay (Section 2.3 and Section 2.4), and AID is the average daily intake specific for each sample, in particular 75–150 g/day for fresh and dried blueberry [34], 0.9 g/day (300 mg capsule × 3 capsules/day) for supplements (the recommended dose reported on the box) and 7 g/day for herbal tea, equal to 1 cup of infusion/day [35].

#### 2.9.2. Estimated Daily Intake of Elements

The estimated daily intake (EDI) of elements was calculated in agreement with Equation (4) reported in the study of Taiwo et al. [36]:

(4) $$EDI=\left(\frac{mg}{kg\times day}\right)=\frac{C\left(\frac{mg}{kg}\right)\times IR\left(\frac{mg}{day}\right)\times ED\left(year\right)\times Ef\left(\frac{day}{year}\right)\times 10^{-6}}{Bw\left(kg\right)\times AT\left(\frac{day}{year}\times year\right)}$$

where C is the concentration of the metal in mg/kg; IR is the ingestion ratio of fruit and herbal teas (150,000 mg/day [34] and 7000 mg/day for adults and children, respectively [35]); ED is the human life exposure (70 and 6 years for adults and children, respectively); Ef is the exposure frequency (365 days/year); Bw is the average body weight (60 and 15 kg for adults and children, respectively); AT is the averaging time, and it is the product of ED and Ef (9125 days for non-carcinogenic elements and 25,550 days for carcinogenic elements).

#### 2.9.3. Non-Carcinogenic Risk

The hazard quotient (HQ) and hazard index (HI) of elements not classified as carcinogenic were calculated in agreement with Equation (5) reported in the study of Taiwo et al. [36]:

(5) $$HQ=\frac{EDI\left(\frac{mg}{kg\;\times \;day}\right)}{RfD\left(\frac{mg}{kg\;\times \;day}\right)};HI=\sum_{i=1}^{n}{HQ}_{i}$$

HQ or HI > 1.0 indicates non-carcinogenic adverse health effects and the presence of a potential health risk; HQ or HI < 1.0 suggests no adverse health effects.

#### 2.9.4. Carcinogenic Risk

The cancer risk (CR) of toxic elements classified as likely carcinogenic and cancerogenic was calculated in agreement with Equation (6) reported in the study of Taiwo et al. [36]:

(6) $$CR=EDI\left(\frac{mg}{kg*day}\right)\times CSF{\left(mg/kg\times day\right)}^{-1}$$

where CSF stands for the cancer slope factor. CR > 1.0 × 10^−4^ indicates possible development of cancer, while CR < 1.0 × 10^−4^ establishes no possible cancer development.

## 3. Results

### 3.1. Optimized Operating Conditions in HPLC-ESI-MS/MS

The optimization of the operational conditions and evaluation of fragmentations for each analyte in mass spectrometry were carried out by infusing standard solutions (10 µg/mL at 10 µL/min) before the analysis in multiple reaction monitoring (MRM) mode with negative ionization. The precursor ions, product ions and electrical parameters are shown in Table 2 and Table 3.

**Figure 1:**
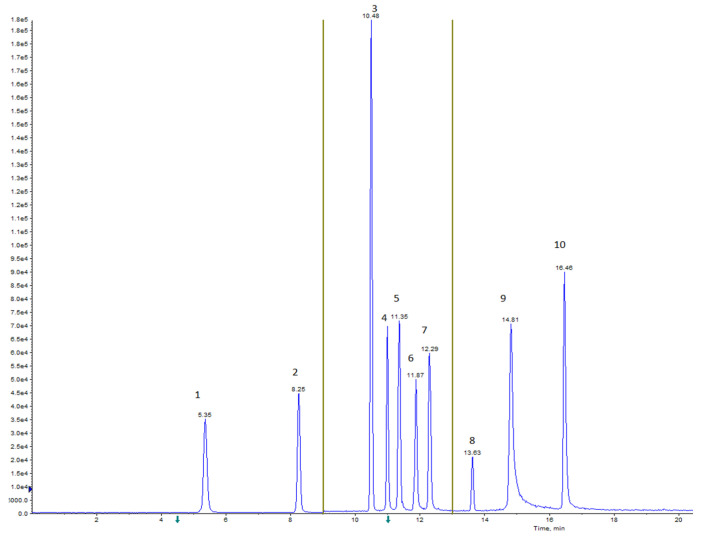
HPLC-ESI-MS/MS chromatogram in MRM mode of polyphenols at a concentration of 5 μg/mL obtained with a Gemini^®^ C-18 column at a flow rate of 300 μL/min by gradient elution reported in Table S2 and with the electrical parameters of the mass spectrometer reported in Table 3, where 1—catechin (5.35 min), 2—epicatechin (8.25 min), 3—astringin (10.48 min), 4—rutin (11.00 min), 5—isoquercitrin (11.35 min), 6—piceatannol (11.87 min), 7—quercitrin (12.29 min), 8—resveratrol (13.63 min), 9–10—luteolin and quercetin (14.81 min), 10–12—hesperetin and kaempferol (16.46 min). The chromatographic run is divided into three acquisition windows (indicated by the arrows in the figure) to improve instrumental resolution. The precursor and product ions chosen are reported in Table 2.

In order to optimize the HPLC polyphenol separation, a gradient elution was carried out using a mobile phase composed of water (H_2_O) and acetonitrile (ACN), both with 0.1% formic acid (HCOOH) (see Table S2). The chromatographic column was set at 40 °C. The injection volume was 8 μL, and the flow rate was 300 μL/min. The twelve polyphenols were eluted within a run time of 22 min, and the retention times are shown in Figure 1.

### 3.2. Validation Parameters

The analytical parameters validated in this study are linearity, limit of detection (LOD), limit of quantification (LOQ) and reproducibility. After evaluating the absence of the matrix effect according to the procedure reported by Simonetti et al. [29], the calibration curves were built in the solvent in a range suited for the analysis of real samples, between 40 µg/L and 5 µg/mL. A linear correlation coefficient (R^2^) higher than 0.99 for all the polyphenols was considered acceptable (see Table 4).

LODs, LOQs and RSD values related to intra-day and inter-day repeatability were calculated as described in Section 2.8 and are reported in Table 5.

LODs were found to range between 20 µg/L and 300 µg/L, while LOQs were found to range between 40 µg/L and 600 µg/L, respectively, for hesperetin and kaempferol. The RSD values related to intra-day and inter-day repeatability were always below 5% and 10%, respectively. The analytical purification procedure scored recoveries between 60% and 100% (calculated as in Section 2.8), depending on the specific polyphenols.

### 3.3. Polyphenolic Profile in Blueberry-Based Samples

All samples underwent pre-treatment, as described in Section 2.2, as well as purification through the modified analytical procedure based on SPE reported by Simonetti et al. [29] and were analyzed in the HPLC-MS/MS system according to the analytical conditions described in the Supplementary Materials. The analysis was performed three times on each sample, and the total flavonoid contents are reported in Table 6.

As shown in Table 6, the herbal teas show a higher total flavonoid content; in particular, the organic blueberry infusion shows the highest content, equal to 8 mg/g. As already noted by other authors [37], it is intuitive to observe that the polyphenol content in the final drink is influenced by the infusion time and temperature. Blueberry-based supplements show an intermediate flavonoid content of 3 mg/g, followed by dried and fresh blueberries, which show fair flavonoid content. Dried blueberries have a much higher flavonoid content compared to fresh blueberries. The difference in the results between dried and fresh blueberries can be attributed to the varying water content present in fresh berries. The reduction in water in dried blueberries could reach up to 80% based on the drying process followed, significantly impacting the composition and concentration of nutrients.

Indeed, when fresh blueberries are dried, the water content in the fruits decreases, leaving bioactive compounds such as polyphenols concentrated in the solid portion. However, in this study, the results between fresh and dried blueberries are independent of each other due to the different initial raw materials.

The objective is to illustrate two potential ways of blueberry consumption based on consumer preferences.

**Figure 2:**
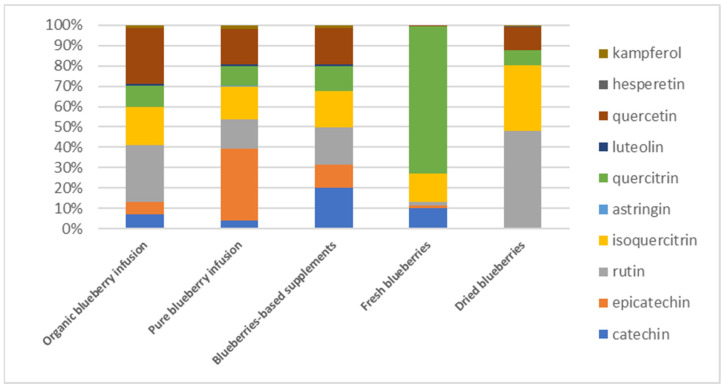
Polyphenolic distribution in the investigated samples.

For a more comprehensive study of the polyphenolic profile, the percentage distribution of the twelve selected compounds for each sample is shown in Figure 2, according to individual compound values reported in Table S3.

The first two histogram columns, left to right, related to herbal teas show a similar polyphenolic composition profile except for a different contribution to the total percentage of rutin and epicatechin. In addition, the occurrence of astringin, present only in very low amounts in the pure blueberry infusion (14.4 µg/g), is probably due to the presence of leaves rich in this flavonoid in this product.

The blueberry-based supplement shows a profile similar to herbal teas and more varied compared to fresh and dried blueberries. Regarding the polyphenolic profile of fresh and dried blueberries, a central role is played by the three quercetin sugar derivatives belonging to the flavonol subclass (quercetin 3-β-D-glucoside, quercetin 3-rhamnoside and quercetin 3-rhamnoglucoside, respectively), corresponding to 88% of total flavonoids, in agreement with the studies of Yao et al. [12].

Specifically, quercitrin stands out in fresh blueberries (72%), while rutin (48%) followed by isoquercetin (32%) stand out in dried blueberries. Quercetin occurs more in dried rather than in fresh berries (around 10% compared to 1%, respectively). Furthermore, hesperetin and kaempferol were identified and quantified in dried blueberries, whereas the same flavonoids were not identified in fresh blueberries (Table S3). Mustafa et al. [38] found a similar distribution profile in fresh blueberries, with quercitrin being the most abundant, followed by isoquercitrin, rutin and quercetin. The polyphenolic compounds present in herbal teas and blueberries, such as rutin, epicatechin, quercetin derivatives, hesperetin and kaempferol, are known for their numerous health benefits. These compounds exhibit potent antioxidant properties, which aid in reducing oxidative stress and inflammation in the body. They also contribute to improved cardiovascular health, better blood sugar regulation and enhanced cognitive function. The presence of these bioactive compounds in both fresh and dried blueberries, as well as in blueberry-based supplements, highlights their potential as functional foods for improving human health and well-being.

### 3.4. TPC and TFC Test Results

As reported in Section 2.4, the TFC test results were quantified on the quercetin calibration curve; however, to enable a comparison between TPC and TFC tests, these results were normalized and expressed in equivalents of gallic acid according to Equation (7):

(7) $$FC(\frac{\mu g}{mL}GAEeq):\frac{{ValueTFC(µg/mL)}_{quercetin}}{4}\times \frac{PMGA}{PMQE}$$

where the TFC value is the test result quantified with respect to quercetin; 4 is the correction factor related to different dilution ratios of TFC and TPC tests; and PMGA and PMQE are, respectively, the molecular weight of gallic acid and quercetin. Table 7 shows the total polyphenolics and flavonoids content normalized in the blueberry-based samples.

As reported in Table 7, the TPC values are, on average, 2–3 times higher than the TFC values. Herbal teas have a higher polyphenolic content in the range of 29–39 mg GAE/g sample, and a significant portion is represented by flavonoids in the range of 10–15 mg GAE/g sample, in agreement with TPC and TFC herbal tea values reported in the literature [24]. Fresh and dried blueberries and blueberry-based supplements have a lower polyphenolic amount in the range of 1–6 mg GAE/g sample and a flavonoids content between 0.5 and 4 mg GAE/g sample, in agreement with TPC and TFC results for blueberries reported in the literature [20,21].

### 3.5. DPPH and ABTS Method Results

Table 8 shows the results of the antioxidant activity test for the blueberry-based samples. Experiments were performed in triplicate, and analyses were repeated three times, with similar results expressed both as mg Trolox/g sample and the percentage of DPPH and ABTS·+ reagent reduction, as described in Section 2.5.

The DPPH and ABTS reduction activity showed the same trend as TPC and TFC assays, with maximum antioxidant activity noted for herbal teas (10–12 mg Trolox/g sample in organic blueberry infusion and 7–12 mg Trolox/g sample in pure blueberry infusion), followed by blueberry-based supplements (7.4 mg Trolox/g sample) and fresh and dried blueberries (0.7–0.8 mg Trolox/g sample), in agreement with the literature [23,24,39]. In particular, blueberry-based supplements showed significantly greater antioxidant activity than fresh and dried blueberries, confirmed by their higher polyphenolic content shown in Table 7.

### 3.6. Intake Based on Nutraceutical Content of Blueberry-Based Samples

Flavonoids are not classified as essential compounds, but the consumption of foods rich in flavonoids may have beneficial effects on health due to their biological and pharmacological properties. However, there is still limited knowledge regarding the mechanisms of these bioactive compounds, and a recommended daily dose (RDA) for each subclass has not yet been established. However, several scientific studies declare a possible beneficial effect with a recommended daily dose range between 250 and 400 mg/day, respecting food seasonality [40]. Starting with the TFC and TPC results, flavonoids daily intake based on the average daily intake of each sample (75–150 g/day for fresh/dried fruit, 0.9 g/day for the supplements and one tea bag/day for herbal tea) was calculated and shown in Table 9.

Table 9 shows how the consumption of 75–150 g/day of dried blueberries leads to a flavonoids intake of between 52 and 105 mg, whereas the same amount of fresh blueberries introduces between 22 and 45 mg/day of flavonoids, clearly due to a higher water content. By contrast, using three capsules of the supplement (300 mg capsule × 3 capsules/day, the recommended dose reported on the box) can lead to an intake of 2.7 mg/day of flavonoids, far below the fresh and dried blueberries. However, it must be considered that the administration of supplement timelines requires a longer time than sporadic intake of the fruit, not considering seasonality. The estimation of the daily intake of flavonoids was also calculated based on the total average content of the twelve polyphenols analyzed in this study (see Table 9). In this case, the results showed the same trend as that calculated for the TFC results and the organic blueberry infusion, confirming the highest total flavonoid intake of 56 mg/day. The TPC data obtained in this study (third column) are lower than those obtained by Gibson et al. [41], who reported that a serving of ripened blueberries (about 125 g) can provide approximately 200–400 mg of TP. In addition, these findings highlight the variability in flavonoid content across different products and the importance of considering both the type and quantity of food consumed to achieve the potential health benefits associated with flavonoids.

### 3.7. Element Levels

The content of essential and trace metals was evaluated to better characterize the blueberry-based samples. In detail, forty elements (Al, As, B, Ba, Be, Bi, Ca, Cd, Ce, Co, Cr, Cs, Cu, Fe, Ga, K, La, Li, Mg, Mn, Mo, Na, Nb, Ni, P, Pb, Rb, Sb, Se, Si, Sn, Sr, Te, Ti, Tl, U, V, W, Zn and Zr) were analyzed, and the results are reported in Supplementary Materials (Table S4). High contents of Mg, K, P and Ca were found in all samples, followed by high concentrations of Fe and Mn. Although Fe and Mn could potentially be toxic if present in excess, the concentrations registered in all samples (in the range of 0.4–10 mg/kg and 0.5–45 mg/kg, respectively) were much lower than the allowable limit in foods (425 and 500 mg/kg, respectively [42]). Zn and Cu were present in smaller quantities (in the range of 0.05–5 mg/kg and 0.02–0.5 mg/kg, respectively); also, in this case, the concentrations registered in all samples were much lower than the allowable limit in foods (60 and 40 mg/kg, respectively [43]). Furthermore, concentrations of toxic or potentially toxic elements were detected in all samples, but these concentrations were below the limit values imposed by Commission Regulation (EU) 2023/915 [44] related to foods and the Codex Herbarum related to herbal teas.

### 3.8. Health Risk Assessment

Since significant metal concentrations were found in all blueberry-based samples, the EDI was calculated to evaluate the metal intake resulting from daily consumption of these foods. In addition, the risk factors (HQ, HI and CR) were calculated to evaluate possible adverse health effects deriving from the consumption of these foods. EDI, HQ, HI and CR were calculated for both adults and young consumers in order to have a more comprehensive overview of human exposure assessment. To calculate these indices, specific average daily intakes for each sample were used, as shown in Table 9. It is important to underline that the range of the fruit ingestion ratio for the EDI calculation and, consequently, for the risk indices was considered 150 g for adults (maximum value) and 75 g (minimum value) for children, based on the different physiological needs.

#### 3.8.1. Estimated Daily Intake (EDI) of Elements in Blueberry-Based Samples

EDI values were calculated as reported in Section 2.9.1, considering metal concentrations in each sample (Section 3.7) and evaluating the maximum daily intake for adults and children; they are reported in Supplementary Materials (Tables S5 and S6). EDI was calculated for those elements that may have adverse effects on human health if present at high concentrations (Al, Cu, Fe, Mn, Ni, Sb, Se, V, Zn) and for elements characterized by high toxicity (As, Cd, Pb). Mn in herbal teas contributed most to EDI (1.08 × 10–2 in organic blueberry infusion and 1.14 × 10^−2^ in pure blueberry infusion). The EDI values for Cu, Mn, Fe, Cd, Pb and As were generally lower than the provisional maximum tolerable daily intake (PMTDI), a safe intake considering the natural occurrence of the substance in food and in drinking water (0.58, 0.016–0.166, 0.8, 0.0012, 0.00357 and 0.00042 mg/kg/day, respectively).

#### 3.8.2. Non-Carcinogenic Risk

**Figure 3:**
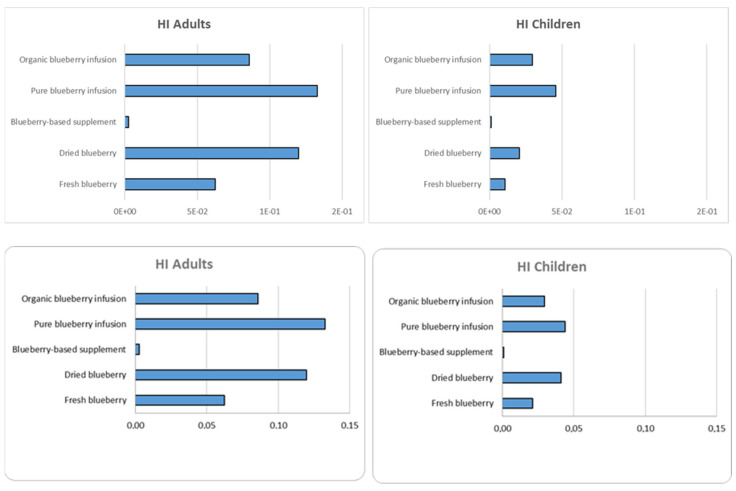
HI index values for non-carcinogenic elements in blueberry-based samples for adults and children.

The non-carcinogenic hazard quotient (HQ) and hazard index (HI) were calculated as reported in Section 2.9.2 for all the metals not classified as carcinogenic but which could have adverse effects on human health if present at high concentrations (Al, Cu, Fe, Mn, Ni, Sb, Se and V). The HQ values are displayed in Supplementary Materials (Tables S7 and S8), and the HI values are reported in Figure 3.

Figure 3 shows the results considering both adult and child exposure; as expected, both trends are comparable. Mn is the most significant contributor to HI in herbal teas; Fe and Al also significantly contribute to HI in the other samples. However, for all samples, the HI values are less than 1, thus showing no non-carcinogenic risk for adults and children.

#### 3.8.3. Carcinogenic Risk

**Figure 4:**
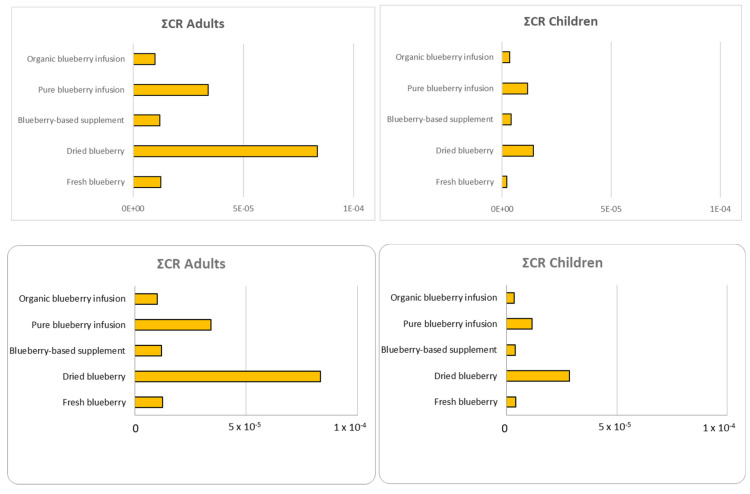
CR values for heavy metals in blueberry-based samples consumed by adults and children.

The cancer risk (CR) was calculated as reported in Section 2.9.3 for elements with high toxicity, such as Pb (classified by IARC as belonging to class 2A), Cd and As (classified by IARC as belonging to class 1), and the results for each metal for both adults and children are reported in Supplementary Materials (Tables S9 and S10). Figure 4 shows the sum of the CR values calculated for each sample for the three heavy metals, with each sample considering both adults and children.

Also, for CR, the results confirm a similar trend both for adults and children. The dried blueberry shows the highest CR values, with As contributing a higher percentage (94%). This finding could be related both to the fruit drying process or to the raw materials used, which could lead to the contamination or concentration of metals in the final product. In particular, As is one of the metals most commonly found in red fruits and vegetables due to its significant presence in the environment. In fact, since As environmental contamination can be attributed to its release via both natural (soil geochemical composition) and anthropogenic activities, it can easily be found especially in plant-based foods [25,30]. However, it should be emphasized that although the dried blueberries showed the highest CR value, it did not exceed the carcinogenic risk limits set by the USEPA (equal to 10–4), which also refers to an intake of maximum dosage of blueberries (150 g every day for a human lifetime exposure of 70 years).

The second sample, which showed a higher CR value, was the pure blueberry infusion. This infusion consists of 50% blueberry leaves and 50% blueberry extract, and the presence of leaves may justify the higher accumulation of heavy metals. In this case, the contribution to the total CR is equally due to both As and Cd percentages (49% and 51%, respectively). Lastly, although the supplement shows the lowest ΣCR values among the analyzed samples, the presence of toxic metals in supplements is widely discussed and can result from various causes, such as contamination or medical traditions according to which the addition of them serves the optimal health balance. Dietary supplement formulations, dosage forms and country of origin are strong determinants of heavy metal contamination in dietary supplement products [45]. The study provides a detailed evaluation of both non-carcinogenic and carcinogenic risks associated with the consumption of blueberry-based products. This dual focus on health risks is relatively novel and offers a more holistic understanding of potential adverse effects. These innovations not only enhance the accuracy of health risk assessments but also provide valuable insights into the safe consumption of blueberry-based products, making this study a significant contribution to food safety research.

## 4. Conclusions

The present work aimed to study the qualitative and quantitative content of flavonoid polyphenols and elements in blueberry-based products. The TPC and TFC optical tests allowed for the calculation of the content of total flavonoids and polyphenols, which appeared to be higher in herbal teas rather than in fresh, dried blueberry and in blueberry-based supplements; the same trend occurred in the DPPH and ABTS reduction activity tests. In particular, the use of HPLC-MS/MS enabled the separation and detection of twelve compounds belonging to different classes of polyphenols. After its validation, the analytical method was successfully applied to real samples after pre-treatment and purification. Ten out of twelve polyphenols were identified and quantified, and it was possible to obtain the relative polyphenolic profile of each sample; herbal teas and blueberry-based supplements showed a varied profile compared to fresh and dried blueberries, where a central role was played by the three sugar derivatives belonging to the flavonol subclass (quercetin 3-β-D-glucoside, quercetin 3-rhamnoside and quercetin 3-rhamnoglucoside, respectively), around 88% of total flavonoids.

Overall, although this is an exploratory study, an interesting aspect that emerged from the study was the variability in polyphenolic profiles between different types of blueberry products. This means that the preparation and preservation methods (such as drying or infusion) can influence the composition and concentration of polyphenols. This could be useful for consumers and nutritionists to guide them toward more informed food choices.

In addition, the estimation of daily intake of flavonoids was calculated based on the TFC and TPC results, assuming a specific intake related to each product. The findings revealed significant variability in flavonoid content across different products. These results underscore the importance of considering both the type and quantity of food consumed to achieve the potential health benefits associated with flavonoids. In parallel, mineral salts content and heavy metals occurrence were evaluated via ICP-MS with the aim of obtaining a more complete nutritional scenario of the real samples. The estimated daily intake (EDI) of metals, the hazard quotient (HQ), the hazard index (HI) and the cancer risk (CR) were calculated to evaluate the actual risk deriving from the intake of these metals.

Risk assessment for heavy metals is crucial, as many plants and fruits, including blueberries, can accumulate toxic metals, such as lead, arsenic and cadmium. Assessing the potential risks from ingestion of these metals (calculating values such as EDI, HQ, HI, CR) is essential to ensure that the benefits of polyphenols are not overshadowed by health risks related to contamination. This is particularly relevant for vulnerable populations, such as children and pregnant women. Currently, research that simultaneously evaluates both polyphenols and metals in the same sample is still limited; yet, such studies could provide valuable data for a more comprehensive understanding of nutritional and health-related aspects. Expanding this research could significantly enhance dietary recommendations by ensuring that polyphenol-rich foods are not only beneficial but also free from toxic metals. Hence, this dual focus on polyphenols and metals is pivotal for advancing public health and nutrition science.

## Figures and Tables

**Table 1:** Composition of the blueberry-based samples tested in this study.

Samples	Type	Composition
Blueberries	Fresh fruit	Fresh blueberries
Blueberries	Dried fruit	Dried blueberries
Blueberry-based supplements	Capsule	A 200 mg capsule of dry blueberry extract (*Vaccinium myrtillus* L.)
Herbal teas	Organic blueberry infusion	Rose hip, blueberry berries and leaves 20%, karcadé, elderberries
	Pure blueberry infusion	Blueberry leaves (*Vaccinium myrtillus* L.) 50% and blueberry berries (*Vaccinium myrtillus* L.) 50%

**Table 2:** Molecular weight, precursor and product ions of polyphenols.

Compound	Molecular Weight (g/mol)	Precursor Ion [M-H]^−^ (*m/z*)	Product Ions (*m/z*) *
Catechin	290	289	109, 125
Epicatechin	290	289	109, 123
Astringin	406	405	243, 201
Rutin	610	609	300, 271
Isoquercitrin	464	463	300, 271
Piceatannol	244	243	201, 159
Quercitrin	448	447	300, 270
Resveratrol	228	227	143, 185
Luteolin	286	285	133, 151
Quercetin	302	301	151, 107
Hesperetin	302	301	164, 151
Kaempferol	286	285	93, 159

* Underlined *m/z* values are the quantifiers.

**Table 3:** Electrical parameters of the mass spectrometer: CUR—curtain gas, CAD—collision gas, IS-V—ion spray voltage, DP—declustering potential, EP—entrance potential, FP—focusing potential, CE—collision energy, CXP—collision cell exit potential.

Compound	CUR (psi)	CAD (psi)	IS-V (V)	DP (V)	EP (V)	FP (V)	CE (eV)	CXP (V)
Catechin	20	3	−4200	−20	−10	−300	−30	−15
Epicatechin	20	3	−4200	−20	−10	−350	−30	−15
Astringin	30	2	−4500	−20	−10	−330	−25	−10
Rutin	20	2	−4300	−20	−10	−330	−48	−10
Isoquercitrin	20	2	−4300	−20	−10	−320	−35	−10
Piceatannol	20	2	−4000	−20	−10	−300	−32	−15
Quercitrin	20	2	−4300	−20	−10	−330	−35	−15
Resveratrol	20	5	−4200	−25	−10	−330	−33	−10
Luteolin	25	2	−4500	−20	−10	−320	−40	−15
Quercetin	20	2	−4500	−20	−10	−300	−30	−15
Hesperetin	25	2	−4300	−20	−10	−350	−35	−15
Kaempferol	20	2	−4500	−20	−10	−330	−48	−15

**Table 4:** Calibration curves and R^2^ of the twelve polyphenols.

Polyphenols	Calibration Curve Equations	R^2^
Catechin	y = 14,500x − 1807.4	0.9981
Epicatechin	y = 15,760x − 1581.7	0.9991
Astringin	y = 154,019x + 11,410	0.9978
Rutin	y = 52,459x – 15,139	0.9964
Isoquercitrin	y = 62,777x – 18,383	0.9989
Piceatannol	y = 14,018x − 1692.2	0.9985
Quercitrin	y = 50,154x − 12,299	0.9954
Resveratrol	y = 11,826x − 118.15	0.9997
Luteolin	y = 54,169x – 14,876	0.9978
Quercetin	y = 16,767x + 2160.7	0.9972
Hesperetin	y = 40,797x + 1938.3	0.9986
Kaempferol	y = 8145.3x − 2010.4	0.9996

**Table 5:** LODs, LOQs, intra-day and inter-day repeatability.

Polyphenols	LOD (μg/mL)	LOQ (μg/mL)	Intra-Day Repeatability (RSD)	Inter-Day Repeatability (RSD)
Catechin	0.08	0.15	1	2
Epicatechin	0.08	0.15	1	2
Astringin	0.04	0.08	3	4
Rutin	0.08	0.15	2	10
Isoquercitrin	0.15	0.31	3	7
Piceatannol	0.31	0.62	5	10
Quercitrin	0.08	0.15	1	2
Resveratrol	0.08	0.15	3	2
Luteolin	0.08	0.15	2	3
Quercetin	0.04	0.08	1	4
Hesperetin	0.02	0.04	5	8
Kaempferol	0.31	0.62	2	4

**Table 6:** Total polyphenolic content in samples.

	Organic Blueberry Infusion	Pure Blueberry Infusion	Blueberry-Based Supplement	Dried Blueberries	Fresh Blueberries
**Σ flavonoids (mg/g)**	8.020 ± 0.010	6.310 ± 0.008	3.570 ± 0.010	1.060 ± 0.002	0.420 ± 0.002

**Table 7:** TPC and TFC results.

Samples	Type	TPC	TFC
		**mg _GAE_/g _sample_**	
Blueberries	Fresh fruit	0.9 ± 0.1	0.40 ± 0.06
Blueberries	Dried fruit	1.2 ± 0.2	0.9 ± 1.1
Blueberry-based supplements	Capsule	5.8 ± 0.8	3.0 ± 1.3
Herbal teas	Organic blueberry infusion	37.1 ± 1.1	13.4 ± 1.3
	Pure blueberry infusion	31.4 ± 2.4	12.2 ± 1.9

**Table 8:** Antioxidant activity results.

Samples	Type	DPPH mg _Trolox_/g _sample_	ABTS	DPPH	ABTS
			% Reduction		
Blueberries	Fresh fruit	0.70 ± 0.01	0.7	35.8	55.2
Blueberries	Dried fruit	0.80 ± 0.02	0.7	33.5	46.9
Blueberry-based supplements	Capsule	7.410 ± 0.002	7.0	34.2	54.1
Herbal teas	Organic blueberry infusion	10.3 ± 0.1	11.7	41.9	70.1
	Pure blueberry infusion	6.3 ± 1.2	11.6	46.1	68.8

**Table 9:** Estimate of flavonoids daily intake.

Samples	Flavonoids Daily Intake (mg/day)	Polyphenols Daily Intake (mg/day)	Average Daily Intake of Each Sample
Fresh blueberries	22–45	67–135	75–150 g/day
Dried blueberries	52–105	90–180	
Blueberry-based supplements	2.7	5.2	0.9 g/day
Organic blueberry infusion	94	260	7 g/day (one tea bag/day)
Pure blueberry infusion	86	220	

## Data Availability

Data will be made available on request.

## Supplementary Materials

The following supporting information can be downloaded at: https://www.mdpi.com/article/10.3390/foods14071210/s1, Figure S1A,B: Flavonoid structures and classification; Table S1: Limits of determination (LODs) and limits of quantification (LOQs) (mg/Kg) of elements; Table S2: Gradient mode used for HPLC polyphenols separation; Table S3: Concentration (μg/g) of the investigated polyphenols in blueberry-based real samples; Table S4: Element concentrations (mg/kg) in blueberry-based samples; Table S5: EDI values calculated for 12 elements (Al, Ni, Cu, Zn, Sb, V, Mn, Fe, Se, Cd, Pb and As) in each samples for adults; Table S6: EDI values calculated for 12 elements (Al, Ni, Cu, Zn, Sb, V, Mn, Fe, Se, Cd, Pb and As) in each sample for children; Table S7: Hazard quotient (HQ) values calculated for metals not classified as carcinogenic (Al, Ni, Cu, Zn, Sb, V, Mn, Fe and Se) for adults; Table S8: Hazard quotient (HQ) values calculated for metals not classified as carcinogenic (Al, Ni, Cu, Zn, Sb, V, Mn, Fe and Se) for children; Table S9: Cancer risk (CR) values calculated for metals with high toxicity (Cd, Pb and As) for adults; Table S10: Cancer risk (CR) values calculated for metals with high toxicity (Cd, Pb and As) for children.
