# Supplementary material for: Exploring the Composition of Blueberry-Based Functional Products: Polyphenolic and Elemental Characterization and Quantification
Source: Foods. 2025 Mar 29;14(7):1210. doi: 10.3390/foods14071210 (PMC11989085; doi:10.3390/foods14071210)
Supplement: Supplementary file 1 [file foods-14-01210-s001.zip › foods-3526084-supplementary.pdf]

SUPPLEMENTARY MATERIAL

Characterization and quantification of polyphenols and elements in blueberry based functional products

Francesca Buiarelli[a], Maria Presutti[a], Maria Luisa Astolfi[a], Carmela Riccardi[b], Donatella Pomata[b] Andrea Fricano[a], Giulia Simonetti[a*], Patrizia Di Filippo[b]

[a] Department of Chemistry, Sapienza University of Rome, P.le Aldo Moro 5-00185 Rome, Italy
[b] Inail DIT-Via Roberto Ferruzzi, 38-00143 Rome, Italy

*Corresponding author giulia.simonetti@uniroma1.it

A. Flavonoid structures and classification

The basic structure mainly refers to a 15-carbon skeleton. rearranged in a configuration of C6-C3-C6. There are two phenyl rings (A and B rings) connected by a skeleton of three carbon atoms. forming a heterocyclic ring (C ring) and it's reported in Fig. S1A.

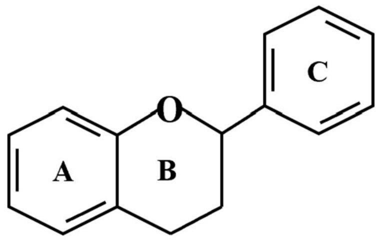

Fig. S1A. Flavonoids basic structure.

Flavonoids are classified into different subclasses (flavones, isoflavones, flavonols, flavanones, flavanonols, flavanols, anthocyanins and chalcones) which differ from each other for the number of hydroxyl groups. degree of saturation. oxidation state. position and number of linked glycosides (Dias et al.. 2021). The classification is reported in Fig. S1B.

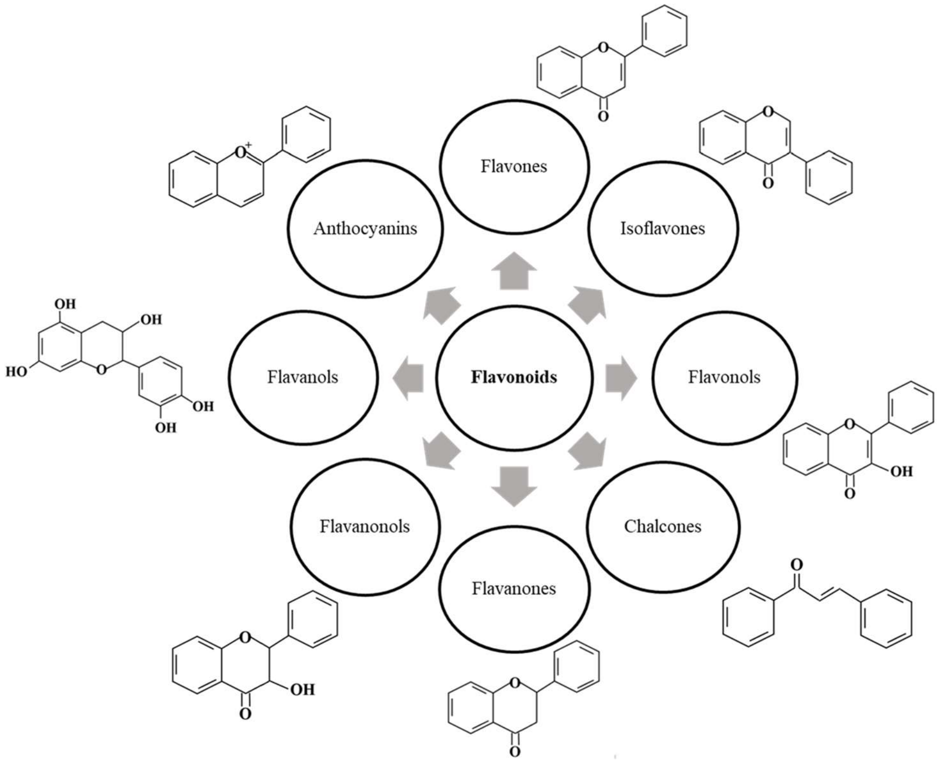

Fig. S1B. Flavonoids classification.

Table S1

LODs (limits of determination) and LOQs (limits of quantification) (mg/Kg) of elements.

Element	LOD	LOQ
**Li**	0.0004	0.001
**Be**	0.000005	0.00002
**B**	0.009	0.03
**Na**	4	13
**Mg**	0.1	0.4
**Al**	0.03	0.1
**Si**	0.2	0.7
**P**	0.02	0.07
**K**	0.1	0.5
**Ti**	0.0003	0.001
**Co**	0.00008	0.0003
**Ni**	0.006	0.02
**Cu**	0.02	0.07
**Zn**	0.1	0.3
**Ga**	0.00001	0.00003
**Rb**	0.0002	0.0007
**Sr**	0.01	0.04
**Zr**	0.00004	0.0001
**Nb**	0.0001	0.0004
**Mo**	0.001	0.004
**Cd**	0.00009	0.0003
**Sn**	0.00007	0.0002
**Sb**	0.00004	0.0001
**Te**	0.00007	0.0002
**Cs**	0.00001	0.00004
**Ba**	0.003	0.009
**La**	0.0001	0.0003
**Ce**	0.0002	0.0006
**W**	0.0007	0.002
**Tl**	0.000004	0.00001
**Pb**	0.0004	0.001
**Bi**	0.0002	0.0006
**U**	0.00001	0.00003
**Ca**	3	12
**V**	0.01	0.03
**Cr**	0.03	0.2
**Mn**	0.01	0.03
**Fe**	0.07	0.2
**As**	0.001	0.002
**Se**	0.001	0.004

Table S2

Gradient mode used for HPLC polyphenols separation.

**Time (minutes)**	**% H_2O +** 0.1% di HCOOH	**%ACN +** 0.1% di HCOOH
0	90	10
2	90	10
20	30	70
22	0	100

Table S3

Concentration (μg/g) of the investigated polyphenols in blueberry-based real samples.

	Blueberry-based samples				
**Polyphenols**	**Organic blueberry infusion**	**Pure blueberry infusion**	**Blueberries-based supplement**	**Dried blueberries**	**Fresh blueberries**
Catechin	557	251	717	ND	41.3
Epicatechin	493	2220	414	ND	6.7
Rutin	2237	911	652	510	7.9
Isoquercitrin	1517	1028	609	338	57.2
Astringin	ND	14.4	ND	ND	1.0
Quercitrin	836	609	449	80.3	303
Luteolin	68.6	64.3	37.5	ND	ND
Quercetin	2190	1093	612	121	2.5
Hesperetin	15.4	9.0	7.7	1.1	ND
Kaempferol	105	111	26.4	6.9	ND
Piceatannol	ND	ND	ND	ND	ND
Resveratrol	ND	ND	ND	ND	ND

**ND: Not Detected*

Table S4. Element concentrations (mg/kg) in blueberry-based samples.

Element	LOD	LOQ	Blueberry-based samples				
			Organic blueberry infusion	Pure blueberry infusion	Blueberry-based supplement	Dried blueberry	Fresh blueberry
**Li**	0.0004	0.001	0.040	0.180	0.082	0.015	0.011
**Be**	0.000005	0.00002	0.00077	0.00410	0.00034	0.00036	0.00001
**B**	0.009	0.03	8.3	74.0	46.0	13.0	4.4
**Na**	4	13	<LOD	<LOD	<LOD	<LOD	38
**Mg**	0.1	0.4	384	677	923	29	12
**Al**	0.03	0.1	1.30	7.20	4.20	0.81	0.51
**Si**	0.2	0.7	9	101	92	22	7
**P**	0.02	0.07	127	312	58	54	17
**K**	0.1	0.5	3670	5800	1150	525	303
**Ti**	0.0003	0.001	0.11	5.20	3.50	0.36	0.37
**Co**	0.00008	0.0003	0.0200	0.0490	0.0011	0.0012	0.0002
**Ni**	0.006	0.02	<LOD	0.350	<LOD	0.015	<LOD
**Cu**	0.02	0.07	0.260	0.580	0.057	0.048	0.024
**Zn**	0.1	0.3	2.20	5.01	0.31	0.42	0.06
**Ga**	0.00001	0.00003	0.0020	0.0100	0.0080	0.0010	0.0010
**Rb**	0.0002	0.0007	4.10	6.50	0.88	0.70	0.23
**Sr**	0.01	0.04	3.7	10.4	1.9	0.3	0.1
**Zr**	0.00004	0.0001	0.007	0.097	0.250	0.009	0.008
**Nb**	0.0001	0.0004	<LOD	0.0079	0.0033	0.0007	0.0004
**Mo**	0.001	0.004	0.011	0.160	0.032	0.007	0.005
**Cd**	0.00009	0.0003	0.0029	0.0099	0.0490	0.0001	0.00002
**Sn**	0.00007	0.0002	0.00590	0.00560	0.00130	0.00077	0.00018
**Sb**	0.00004	0.0001	<LOD	0.00280	0.00027	0.00064	0.00017
**Te**	0.00007	0.0002	0.00029	0.00063	<LOD	0.00007	0.00001
**Cs**	0.00001	0.00004	0.0610	0.0820	0.0040	0.0110	0.0010
**Ba**	0.003	0.009	4.1	13.0	12.0	1.1	1.4
**La**	0.0001	0.0003	0.0190	0.0760	0.0014	0.0012	0.0001
**Ce**	0.0002	0.0006	0.020	0.140	0.051	0.010	0.004
**W**	0.0007	0.002	0.016	0.400	0.020	0.006	0.006
**Tl**	0.000004	0.00001	0.00053	0.00130	0.00110	0.00024	0.00004
**Pb**	0.0004	0.001	0.022	0.047	0.028	0.003	0.001
**Bi**	0.0002	0.0006	<LOD	<LOD	0.0013	<LOD	<LOD
**U**	0.00001	0.00003	0.00068	0.00170	0.00140	0.00021	0.00008
**Ca**	3	12	407	1162	91	53	10
**V**	0.01	0.03	0.04	0.100	0.120	0.010	0.007
**Cr**	0.03	0.2	ND	<LOD	<LOD	<LOD	<LOD
**Mn**	0.01	0.03	33.0	44.0	3.3	1.2	0.8
**Fe**	0.07	0.2	0.39	10.7	8.4	1.0	0.5
**As**	0.001	0.002	0.028	0.095	0.038	0.021	0.003
**Se**	0.001	0.004	0.022	0.010	<LOD	<LOD	<LOD

LOD, limit of determination; LOQ, limit of quantification. Standard deviation <10% of result value.

Tables S5

EDI values calculated for 12 elements (Al, Ni, Cu, Zn, Sb, V, Mn, Fe, Se, Cd, Pb and As) in each samples for adults.

Element	Organic blueberry infusion	Pure blueberry infusion	Blueberry-based supplement	Dried blueberry	Fresh blueberry
**Al**	4.3E-04	2.4E-03	1.8E-04	5.7E-03	3.6E-03
**Ni**	ND	1.1E-04	ND	1.0E-04	ND
**Cu**	8.6E-05	1.9E-04	2.4E-06	3.3E-04	1.7E-04
**Zn**	7.2E-04	1.6E-03	1.3E-05	2.9E-03	4.0E-04
**Sb**	ND	9.2E-07	1.1E-08	4.5E-06	1.2E-06
**V**	1.3E-05	3.3E-05	5.0E-06	6.8E-05	4.6E-05
**Mn**	1.1E-02	1.4E-02	1.4E-04	8.5E-03	5.5E-03
**Fe**	1.3E-04	3.5E-03	3.5E-04	7.3E-03	3.7E-03
**Se**	7.2E-06	3.3E-06	ND	ND	ND
**Cd**	3.5E-07	1.2E-06	7.5E-07	3.3E-07	4.3E-08
**Pb**	2.5E-06	5.5E-06	4.2E-07	8.3E-06	3.0E-06
**As**	3.2E-06	1.1E-05	5.6E-07	5.2E-05	7.9E-06

Table S6

EDI values calculated for 12 elements (Al, Ni, Cu, Zn, Sb, V, Mn, Fe, Se, Cd, Pb and As) in each samples for children.

Element	Organic blueberry infusion	Pure blueberry infusion	Blueberry-based supplement	Dried blueberry	Fresh blueberry
**Al**	1.5E-04	8.1E-04	6.1E-05	9.7E-04	6.2E-04
**Ni**	ND	3.9E-05	ND	1.8E-05	ND
**Cu**	2.9E-05	6.5E-05	8.2E-07	5.7E-05	2.8E-05
**Zn**	2.5E-04	5.6E-04	4.4E-06	5.0E-04	6.9E-05
**Sb**	ND	3.2E-07	3.8E-09	7.7E-07	2.0E-07
**V**	4.5E-06	1.1E-05	1.7E-06	1.2E-05	7.9E-06
**Mn**	3.7E-03	4.9E-03	4.7E-05	1.4E-03	9.4E-04
**Fe**	4.4E-05	1.2E-03	1.2E-04	1.2E-03	6.4E-04
**Se**	2.5E-06	1.1E-06	ND	ND	ND
**Cd**	1.2E-07	3.9E-07	2.6E-07	5.7E-08	7.4E-09
**Pb**	8.7E-07	1.9E-06	1.4E-07	1.4E-06	5.2E-07
**As**	1.1E-06	3.8E-06	1.9E-07	8.9E-06	1.3E-06

Table S7

Hazard Quotient (HQ) values calculated for metals not classified as carcinogenic (Al, Ni, Cu, Zn, Sb, V, Mn, Fe and Se) for adults.

Element	Organic blueberry infusion	Pure blueberry infusion	Blueberry-based supplement	Dried blueberry	Fresh blueberry
**Al**	4.3E-04	2.4E-03	1.8E-04	5.7E-03	3.6E-03
**Ni**	ND	5.7E-03	ND	5.1E-03	ND
**Cu**	2.2E-03	4.7E-03	5.9E-05	8.3E-03	4.2E-03
**Zn**	2.4E-03	5.4E-03	4.3E-05	9.8E-03	1.3E-03
**Sb**	ND	2.3E-03	2.8E-05	1.1E-02	3.0E-03
**V**	2.6E-03	6.5E-03	1.0E-03	1.3E-02	9.2E-03
**Mn**	7.7E-02	1.0E-01	9.8E-04	6.1E-02	3.9E-02
**Fe**	1.8E-04	4.9E-03	5.0E-04	1.0E-02	5.3E-03
**Se**	1.4E-03	6.6E-04	ND	ND	ND

Table S8

Hazard Quotient (HQ) values calculated for metals not classified as carcinogenic (Al, Ni, Cu, Zn, Sb, V, Mn, Fe and Se) for children.

Element	Organic blueberry infusion	Pure blueberry infusion	Blueberry-based supplement	Dried blueberry	Fresh blueberry
**Al**	9.1E-06	5.0E-05	3.8E-06	6.0E-05	3.8E-05
**Ni**	ND	1.7E-03	ND	8.8E-04	ND
**Cu**	7.4E-04	1.6E-03	2.0E-05	1.4E-03	7.2E-04
**Zn**	8.2E-04	1.9E-03	1.5E-05	1.7E-03	2.3E-04
**Sb**	ND	7.9E-04	9.6E-06	1.9E-03	5.2E-04
**V**	9.1E-04	2.2E-03	3.4E-04	2.3E-03	1.6E-03
**Mn**	2.6E-02	3.5E-02	3.3E-04	1.0E-02	6.7E-03
**Fe**	6.3E-05	1.7E-03	1.7E-04	1.8E-03	9.1E-04
**Se**	4.9E-04	2.2E-04	ND	ND	ND

Table S9

Cancer Risk (CR) values calculated for metals with high toxicity (Cd, Pb and As) for adults.

Element	Organic blueberry infusion	Pure blueberry infusion	Blueberry-based supplement	Dried blueberry	Fresh blueberry
**Cd**	5.2E-06	1.7E-05	1.1E-05	5.0E-06	6.5E-07
**Pb**	2.2E-08	4.7E-08	3.5E-09	7.1E-08	2.6E-08
**As**	4.8E-06	1.7E-05	8.4E-07	7.8E-05	1.2E-05

Table S10

Cancer Risk (CR) values calculated for metals with high toxicity (Cd, Pb and As) for children.

Element	Organic blueberry infusion	Pure blueberry infusion	Blueberry-based supplement	Dried blueberry	Fresh blueberry
**Cd**	1.8E-06	5.9E-06	3.8E-06	5.7E-08	7.4E-09
**Pb**	7.4E-09	1.6E-08	1.2E-09	1.4E-06	5.2E-07
**As**	1.6E-06	5.7E-06	2.9E-07	8.9E-06	1.3E-06
